# LONELINESS AMONG UNDER-REPRESENTED OLDER ADULTS IN THE UK: A STUDY OF MINORITY ETHNIC AND LGBTQ+ POPULATIONS

**DOI:** 10.1093/geroni/igad104.3453

**Published:** 2023-12-21

**Authors:** Christina Victor, Isla Rippon

**Affiliations:** Brunel University London, Uxbridge, England, United Kingdom; Brunel University London, Uxbridge, England, United Kingdom

## Abstract

Internationally loneliness has been identified as a major public health problem. Although there is a substantial body of research about loneliness in older adults in the UK, there is a significant evidence gap reporting experiences of loneliness of older people from ethnic minorities and those who identify as lesbian, gay, or bisexual and transgender (LGBT). These two groups, under-represented in UK gerontological research, are included in our recently funded project, Socially Inclusive Ageing across the Lifecourse. In this poster we explicitly focus upon the experiences of loneliness for older adults, aged 50+, from the LGBTQ+ and minority ethnic communities. Using wave 9 data from the UK Household Longitudinal Study (UKHLS/Understanding Society) we measured loneliness using the three-item UCLA scale with a score of 6+ out of 9 defining loneliness. Of our total sample of 16,805 who completed the loneliness measure, 1.5% of respondents identified as LGB and 5.4% as Asian, 2.9% as black and 1.5% as other or mixed ethnicity. Overall, 21.7% of the population aged 50+ were lonely. Participants from a black, Asian or other ethnic minority reported higher loneliness than white respondents: 25.8%, 29.6%, 31.0% and 21.0% respectively. Respondents identifying as gay or lesbian (29.1%) or as bisexual (35.2%) reported greater loneliness in comparison to those who identified as heterosexual (21.3%). Our study is novel and timely in demonstrating the higher prevalence of loneliness in two under-represented groups of older adults with the potential consequences this may have for their health and wellbeing in later life.

